# Ethyl 3,4-dimethyl-5-[(*E*)-(phenyl­imino)­meth­yl]-1*H*-pyrrole-2-carboxyl­ate

**DOI:** 10.1107/S1600536810022051

**Published:** 2010-06-16

**Authors:** Wei-Na Wu, Lei Yang, Xiao-Xia Li, Bao-Feng Qin, Qiu-Fen Wang

**Affiliations:** a Department of Physics and Chemistry, Henan Polytechnic University, Jiaozuo 454000, People’s Republic of China; b Institute of Functional Materials, Jiangxi University of Finance & Economics, Nanchang330013, People’s Republic of China; c Lanzhou Institute of Chemical Physics, Chinese Academy of Sciences, Lanzhou 730000, People’s Republic of China

## Abstract

In the title compound, C_16_H_18_N_2_O_2_, the mol­ecule adopts an *E* conformation about the C=N double bond. The dihedral angle between the pyrrole and phenyl rings is 41.55 (8)°. In the crystal structure, pairs of inter­molecular N—H⋯O hydrogen bonds link the mol­ecules into centrosymmetric dimers. In the dimer, the two pyrrole rings are almost coplanar and the two phenyl rings are parallel to each other.

## Related literature

For the structure of 5-formyl-3,4-dimethyl-1*H*-pyrrole-2-carboxyl­ate, see Wu *et al.* (2009 ▶). For the similar structure of ethyl 5-[(2,3-dimethyl-5-oxo-1-phenyl-2,5-dihydro-1*H*-pyra­zol-4-yl)imino­meth­yl]-3,4-dimethyl-1*H*-pyrrole-2-carboxyl­ate, see Wang *et al.* (2009 ▶). For the coordination abilities for metal ions of pyrrol-2-yl­methyl­ene­amine ligands, see: Wang *et al.* (2010 ▶); Yang *et al.* (2003 ▶).
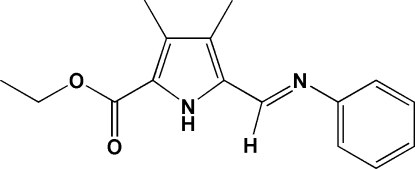


## Experimental

### 

#### Crystal data

C_16_H_18_N_2_O_2_
*M* *_r_* = 270.32
Monoclinic, 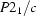

*a* = 12.5463 (7) Å
*b* = 14.6525 (9) Å
*c* = 8.4490 (5) Å
β = 105.042 (3)°
*V* = 1500.00 (15) Å^3^
*Z* = 4
Mo *K*α radiation
μ = 0.08 mm^−1^
*T* = 296 K
0.35 × 0.26 × 0.18 mm

#### Data collection

Bruker SMART CCD diffractometer
Absorption correction: multi-scan (*SADABS*; Sheldrick, 1996 ▶) *T*_min_ = 0.975, *T*_max_ = 0.986
12405 measured reflections
3413 independent reflections
2078 reflections with *I* > 2σ(*I*)
*R*_int_ = 0.030

#### Refinement

*R*[*F* ^2^ > 2σ(*F* ^2^)] = 0.044
*wR*(*F* ^2^) = 0.144
*S* = 1.01
3413 reflections
184 parameters
H-atom parameters constrained
Δρ_max_ = 0.20 e Å^−3^
Δρ_min_ = −0.16 e Å^−3^

### 

Data collection: *SMART* (Bruker, 1997 ▶); cell refinement: *SAINT* (Bruker, 1997 ▶); data reduction: *SAINT*; program(s) used to solve structure: *SHELXS97* (Sheldrick, 2008 ▶); program(s) used to refine structure: *SHELXL97* (Sheldrick, 2008 ▶); molecular graphics: *SHELXTL* (Sheldrick, 2008 ▶); software used to prepare material for publication: *SHELXTL*.

## Supplementary Material

Crystal structure: contains datablocks I, global. DOI: 10.1107/S1600536810022051/gw2080sup1.cif

Structure factors: contains datablocks I. DOI: 10.1107/S1600536810022051/gw2080Isup2.hkl

Additional supplementary materials:  crystallographic information; 3D view; checkCIF report

## Figures and Tables

**Table 1:** Hydrogen-bond geometry (Å, °)

*D*—H⋯*A*	*D*—H	H⋯*A*	*D*⋯*A*	*D*—H⋯*A*
N2—H2*A*⋯O3^i^	0.86	2.06	2.8883 (18)	162

Symmetry code: (i) 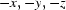.

**Fig. 1.**
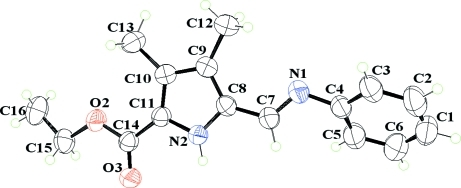
The molecular structure shown with 50% probability displacement ellipsoids.

**Fig. 2.**
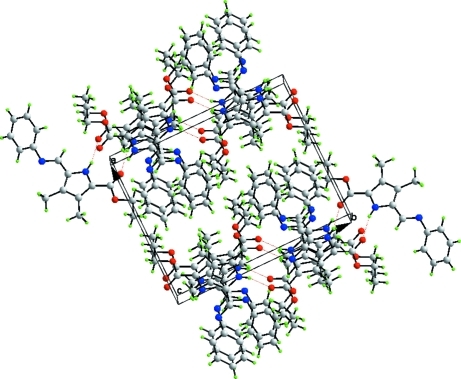
Crystal packing of the title compound showing the dimers formed by hydrogen bonds (dashed lines).

## supplementary crystallographic information

### Comment

Pyrrol-2-ylmethyleneamine ligands have attracted much recent attention due to their excellent coordination abilities for metal ions (Yang *et al.*, 2006 & Wang *et al.*, 2010). As part of our ongoing search for a biologically active material, the title compound was synthesized and characterized by X-ray diffraction.

In the title compound, all the bond lengths are comparable with those observed in the other similar compound (Wang *et al.*, 2009). The molecule adopts an E configuration at the C=N double bond. The dihedral angle between pyrrole ring (N2/C8–C11, r.m.s. deviation 0.0035 Å) and phenyl ring (C1–C6, r.m.s. deviation 0.0036 Å) is 41.55 (8)°. In the crystal, the molecules are linked into a centrosymmetric dimer by two intermolecular N—H···O hydrogen bonds, forming a *R*_2_^2^(10) ring motif (Table1, Fig. 2). In the dimer, the two pyrrole rings are almost coplanar (r.m.s. deviation 0.028 Å) and the two phenyl rings are parallel with each other. The crystal packing is further stabilized by the stacking between the C=N with the adjacent pyrrole ring, with centroid–centroid distances of 3.642 Å.

### Experimental

A quantity of aniline (0.186 g, 2 mmol) was dissolved in ethanol (10 ml), then an ethanol solution (10 ml) containing ethyl 5-formyl-3,4-dimethyl-1*H*-pyrrole-2-carboxylate (0.39 g, 2 mmol) was added dropwise at room temperature. After stirring for 4 h, the mixture was filtered and set aside to crystallize at room temperature for several days, giving yellow block crystals.

### Refinement

All H atoms were placed in calculated positions, with C—H = 0.93–0.97 Å and N—H = 0.86 Å, and were thereafter treated as riding, with *U*_iso_(H) values of 1.5Ueq(C) for methyl groups and 1.2Ueq(C,*N*) for others.

### Crystal data

**Table:**

C_16_H_18_N_2_O_2_	*F*(000) = 576
*M**_r_* = 270.32	*D*_x_ = 1.197 Mg m^−^^3^
Monoclinic, *P*2_1_/*c*	Mo *K*α radiation, λ = 0.71073 Å
Hall symbol: -P 2ybc	Cell parameters from 5515 reflections
*a* = 12.5463 (7) Å	θ = 2.2–26.2°
*b* = 14.6525 (9) Å	µ = 0.08 mm^−^^1^
*c* = 8.4490 (5) Å	*T* = 296 K
β = 105.042 (3)°	Block, yellow
*V* = 1500.00 (15) Å^3^	0.35 × 0.26 × 0.18 mm
*Z* = 4	

### Data collection

**Table:**

Bruker SMART CCD diffractometer	3413 independent reflections
Radiation source: fine-focus sealed tube	2078 reflections with *I* > 2σ(*I*)
graphite	*R*_int_ = 0.030
phi and ω scans	θ_max_ = 27.6°, θ_min_ = 1.7°
Absorption correction: multi-scan (*SADABS*; Sheldrick, 1996)	*h* = −14→16
*T*_min_ = 0.975, *T*_max_ = 0.986	*k* = −14→19
12405 measured reflections	*l* = −10→8

### Refinement

**Table:**

Refinement on *F*^2^	Primary atom site location: structure-invariant direct methods
Least-squares matrix: full	Secondary atom site location: difference Fourier map
*R*[*F*^2^ > 2σ(*F*^2^)] = 0.044	Hydrogen site location: inferred from neighbouring sites
*wR*(*F*^2^) = 0.144	H-atom parameters constrained
*S* = 1.01	*w* = 1/[σ^2^(*F*_o_^2^) + (0.0645*P*)^2^ + 0.3198*P*] where *P* = (*F*_o_^2^ + 2*F*_c_^2^)/3
3413 reflections	(Δ/σ)_max_ = 0.007
184 parameters	Δρ_max_ = 0.20 e Å^−^^3^
0 restraints	Δρ_min_ = −0.16 e Å^−^^3^

### Special details

**Table:**

Geometry. All e.s.d.'s (except the e.s.d. in the dihedral angle between two l.s. planes) are estimated using the full covariance matrix. The cell e.s.d.'s are taken into account individually in the estimation of e.s.d.'s in distances, angles and torsion angles; correlations between e.s.d.'s in cell parameters are only used when they are defined by crystal symmetry. An approximate (isotropic) treatment of cell e.s.d.'s is used for estimating e.s.d.'s involving l.s. planes.
Refinement. Refinement of *F*^2^ against ALL reflections. The weighted *R*-factor *wR* and goodness of fit *S* are based on *F*^2^, conventional *R*-factors *R* are based on *F*, with *F* set to zero for negative *F*^2^. The threshold expression of *F*^2^ > σ(*F*^2^) is used only for calculating *R*-factors(gt) *etc*. and is not relevant to the choice of reflections for refinement. *R*-factors based on *F*^2^ are statistically about twice as large as those based on *F*, and *R*- factors based on ALL data will be even larger.

### Fractional atomic coordinates and isotropic or equivalent isotropic displacement parameters (Å^2^)

**Table:**

	*x*	*y*	*z*	*U*_iso_*/*U*_eq_	
N2	0.02009 (11)	0.14614 (9)	0.03549 (16)	0.0474 (3)	
H2A	−0.0130	0.0983	−0.0125	0.057*	
O2	0.24807 (9)	0.06709 (8)	0.35169 (14)	0.0576 (3)	
O3	0.12317 (10)	−0.01491 (9)	0.17093 (15)	0.0612 (4)	
C14	0.15990 (13)	0.05888 (12)	0.22476 (19)	0.0462 (4)	
C11	0.11351 (13)	0.14570 (11)	0.16129 (19)	0.0444 (4)	
N1	−0.15004 (12)	0.33049 (10)	−0.16873 (18)	0.0545 (4)	
C8	−0.01284 (13)	0.23366 (11)	−0.0027 (2)	0.0466 (4)	
C10	0.14228 (13)	0.23604 (11)	0.20337 (19)	0.0458 (4)	
C4	−0.25266 (15)	0.33933 (12)	−0.2860 (2)	0.0543 (5)	
C7	−0.11212 (14)	0.25067 (12)	−0.1305 (2)	0.0514 (4)	
H7	−0.1500	0.2012	−0.1874	0.062*	
C9	0.06264 (13)	0.29161 (11)	0.1002 (2)	0.0465 (4)	
C12	0.05849 (16)	0.39340 (12)	0.1001 (2)	0.0619 (5)	
H12A	0.0308	0.4150	−0.0102	0.093*	
H12B	0.1313	0.4172	0.1452	0.093*	
H12C	0.0106	0.4135	0.1652	0.093*	
C3	−0.26627 (17)	0.41010 (14)	−0.3976 (2)	0.0634 (5)	
H3	−0.2072	0.4482	−0.3985	0.076*	
C13	0.24063 (14)	0.26959 (13)	0.3311 (2)	0.0606 (5)	
H13A	0.2367	0.3347	0.3399	0.091*	
H13B	0.3067	0.2533	0.3006	0.091*	
H13C	0.2416	0.2422	0.4347	0.091*	
C2	−0.3674 (2)	0.42436 (17)	−0.5076 (3)	0.0782 (7)	
H2	−0.3756	0.4714	−0.5838	0.094*	
C15	0.29547 (16)	−0.01538 (14)	0.4354 (2)	0.0635 (5)	
H15A	0.3295	−0.0515	0.3656	0.076*	
H15B	0.2388	−0.0519	0.4644	0.076*	
C16	0.37962 (17)	0.01386 (17)	0.5854 (3)	0.0793 (6)	
H16A	0.4327	0.0528	0.5553	0.119*	
H16B	0.4163	−0.0389	0.6416	0.119*	
H16C	0.3442	0.0465	0.6562	0.119*	
C5	−0.34233 (16)	0.28429 (15)	−0.2859 (3)	0.0727 (6)	
H5	−0.3348	0.2364	−0.2116	0.087*	
C1	−0.4555 (2)	0.37029 (19)	−0.5058 (3)	0.0869 (7)	
H1	−0.5238	0.3809	−0.5789	0.104*	
C6	−0.44248 (18)	0.30041 (18)	−0.3958 (3)	0.0893 (7)	
H6	−0.5023	0.2631	−0.3950	0.107*	

### Atomic displacement parameters (Å^2^)

**Table:**

	*U*^11^	*U*^22^	*U*^33^	*U*^12^	*U*^13^	*U*^23^
N2	0.0494 (8)	0.0418 (8)	0.0461 (8)	0.0011 (6)	0.0038 (6)	−0.0006 (6)
O2	0.0557 (7)	0.0545 (8)	0.0523 (7)	0.0022 (6)	−0.0044 (6)	0.0051 (6)
O3	0.0666 (8)	0.0467 (8)	0.0594 (8)	0.0035 (6)	−0.0030 (6)	−0.0027 (6)
C14	0.0456 (9)	0.0511 (11)	0.0405 (9)	0.0002 (8)	0.0086 (7)	−0.0011 (7)
C11	0.0459 (9)	0.0457 (9)	0.0396 (9)	−0.0005 (7)	0.0075 (7)	0.0011 (7)
N1	0.0567 (9)	0.0494 (9)	0.0542 (9)	0.0074 (7)	0.0083 (7)	0.0051 (7)
C8	0.0506 (9)	0.0433 (9)	0.0460 (9)	0.0041 (7)	0.0128 (7)	0.0036 (7)
C10	0.0492 (9)	0.0479 (10)	0.0420 (9)	−0.0043 (7)	0.0149 (7)	−0.0020 (7)
C4	0.0579 (10)	0.0494 (10)	0.0528 (11)	0.0119 (8)	0.0092 (8)	0.0005 (8)
C7	0.0540 (10)	0.0481 (10)	0.0496 (10)	0.0036 (8)	0.0089 (8)	0.0022 (8)
C9	0.0512 (9)	0.0446 (9)	0.0465 (9)	−0.0008 (7)	0.0176 (8)	0.0013 (7)
C12	0.0680 (12)	0.0455 (10)	0.0714 (13)	−0.0028 (9)	0.0164 (10)	−0.0009 (9)
C3	0.0763 (13)	0.0590 (12)	0.0550 (11)	0.0166 (10)	0.0170 (10)	0.0081 (9)
C13	0.0593 (11)	0.0601 (12)	0.0583 (12)	−0.0110 (9)	0.0079 (9)	−0.0053 (9)
C2	0.0990 (17)	0.0783 (15)	0.0530 (12)	0.0344 (14)	0.0118 (12)	0.0095 (11)
C15	0.0636 (11)	0.0639 (12)	0.0568 (11)	0.0111 (9)	0.0046 (9)	0.0116 (9)
C16	0.0634 (12)	0.1025 (18)	0.0616 (13)	0.0050 (12)	−0.0025 (10)	0.0135 (12)
C5	0.0632 (12)	0.0629 (13)	0.0847 (15)	0.0045 (10)	0.0058 (11)	0.0142 (10)
C1	0.0742 (15)	0.0951 (19)	0.0755 (16)	0.0244 (14)	−0.0093 (12)	−0.0066 (13)
C6	0.0633 (13)	0.0848 (17)	0.106 (2)	0.0012 (12)	−0.0026 (13)	−0.0002 (15)

### Geometric parameters (Å, °)

**Table:**

N2—C8	1.360 (2)	C12—H12C	0.9600
N2—C11	1.363 (2)	C3—C2	1.381 (3)
N2—H2A	0.8600	C3—H3	0.9300
O2—C14	1.3313 (18)	C13—H13A	0.9600
O2—C15	1.448 (2)	C13—H13B	0.9600
O3—C14	1.216 (2)	C13—H13C	0.9600
C14—C11	1.443 (2)	C2—C1	1.363 (3)
C11—C10	1.394 (2)	C2—H2	0.9300
N1—C7	1.272 (2)	C15—C16	1.487 (3)
N1—C4	1.412 (2)	C15—H15A	0.9700
C8—C9	1.395 (2)	C15—H15B	0.9700
C8—C7	1.443 (2)	C16—H16A	0.9600
C10—C9	1.403 (2)	C16—H16B	0.9600
C10—C13	1.496 (2)	C16—H16C	0.9600
C4—C3	1.382 (2)	C5—C6	1.375 (3)
C4—C5	1.384 (3)	C5—H5	0.9300
C7—H7	0.9300	C1—C6	1.364 (3)
C9—C12	1.492 (2)	C1—H1	0.9300
C12—H12A	0.9600	C6—H6	0.9300
C12—H12B	0.9600		
			
C8—N2—C11	109.65 (13)	C4—C3—H3	119.9
C8—N2—H2A	125.2	C2—C3—H3	119.9
C11—N2—H2A	125.2	C10—C13—H13A	109.5
C14—O2—C15	117.92 (14)	C10—C13—H13B	109.5
O3—C14—O2	122.44 (15)	H13A—C13—H13B	109.5
O3—C14—C11	124.58 (15)	C10—C13—H13C	109.5
O2—C14—C11	112.98 (14)	H13A—C13—H13C	109.5
N2—C11—C10	107.94 (14)	H13B—C13—H13C	109.5
N2—C11—C14	118.44 (14)	C1—C2—C3	120.7 (2)
C10—C11—C14	133.60 (15)	C1—C2—H2	119.6
C7—N1—C4	118.36 (15)	C3—C2—H2	119.6
N2—C8—C9	108.10 (14)	O2—C15—C16	106.66 (17)
N2—C8—C7	119.37 (15)	O2—C15—H15A	110.4
C9—C8—C7	132.53 (16)	C16—C15—H15A	110.4
C11—C10—C9	107.29 (14)	O2—C15—H15B	110.4
C11—C10—C13	127.36 (15)	C16—C15—H15B	110.4
C9—C10—C13	125.34 (16)	H15A—C15—H15B	108.6
C3—C4—C5	118.75 (18)	C15—C16—H16A	109.5
C3—C4—N1	118.45 (17)	C15—C16—H16B	109.5
C5—C4—N1	122.62 (17)	H16A—C16—H16B	109.5
N1—C7—C8	122.82 (16)	C15—C16—H16C	109.5
N1—C7—H7	118.6	H16A—C16—H16C	109.5
C8—C7—H7	118.6	H16B—C16—H16C	109.5
C8—C9—C10	107.02 (14)	C6—C5—C4	120.0 (2)
C8—C9—C12	126.15 (15)	C6—C5—H5	120.0
C10—C9—C12	126.84 (15)	C4—C5—H5	120.0
C9—C12—H12A	109.5	C2—C1—C6	119.4 (2)
C9—C12—H12B	109.5	C2—C1—H1	120.3
H12A—C12—H12B	109.5	C6—C1—H1	120.3
C9—C12—H12C	109.5	C1—C6—C5	121.0 (2)
H12A—C12—H12C	109.5	C1—C6—H6	119.5
H12B—C12—H12C	109.5	C5—C6—H6	119.5
C4—C3—C2	120.1 (2)		
			
C15—O2—C14—O3	−5.1 (2)	C9—C8—C7—N1	−1.8 (3)
C15—O2—C14—C11	174.30 (15)	N2—C8—C9—C10	−0.44 (18)
C8—N2—C11—C10	−0.93 (18)	C7—C8—C9—C10	178.79 (17)
C8—N2—C11—C14	177.75 (15)	N2—C8—C9—C12	179.75 (15)
O3—C14—C11—N2	2.1 (3)	C7—C8—C9—C12	−1.0 (3)
O2—C14—C11—N2	−177.28 (13)	C11—C10—C9—C8	−0.12 (18)
O3—C14—C11—C10	−179.64 (17)	C13—C10—C9—C8	178.52 (15)
O2—C14—C11—C10	1.0 (3)	C11—C10—C9—C12	179.69 (16)
C11—N2—C8—C9	0.85 (18)	C13—C10—C9—C12	−1.7 (3)
C11—N2—C8—C7	−178.50 (14)	C5—C4—C3—C2	−0.8 (3)
N2—C11—C10—C9	0.63 (18)	N1—C4—C3—C2	−176.03 (17)
C14—C11—C10—C9	−177.76 (18)	C4—C3—C2—C1	1.3 (3)
N2—C11—C10—C13	−177.97 (15)	C14—O2—C15—C16	−171.10 (15)
C14—C11—C10—C13	3.6 (3)	C3—C4—C5—C6	0.2 (3)
C7—N1—C4—C3	−141.86 (17)	N1—C4—C5—C6	175.21 (19)
C7—N1—C4—C5	43.1 (3)	C3—C2—C1—C6	−1.2 (3)
C4—N1—C7—C8	−174.75 (16)	C2—C1—C6—C5	0.6 (4)
N2—C8—C7—N1	177.38 (16)	C4—C5—C6—C1	−0.1 (4)

### Hydrogen-bond geometry (Å, °)

**Table:**

*D*—H···*A*	*D*—H	H···*A*	*D*···*A*	*D*—H···*A*
N2—H2A···O3^i^	0.86	2.06	2.8883 (18)	162

Symmetry codes: (i) −*x*, −*y*, −*z*.
